# Frequency and Factors Associated With Adverse Events Among Multi-Drug Resistant Tuberculosis Patients in Pakistan: A Retrospective Study

**DOI:** 10.3389/fmed.2021.790718

**Published:** 2022-03-01

**Authors:** Muhammad Atif, Waqar Ahmed, Muhammad Nouman Iqbal, Nafees Ahmad, Wajiha Ahmad, Iram Malik, Yaser Mohammed Al-Worafi

**Affiliations:** ^1^Department of Pharmacy Practice, Faculty of Pharmacy, The Islamia University of Bahawalpur, Bahawalpur, Pakistan; ^2^Sardarpur Rural Health Center, Khanewal, Pakistan; ^3^Department of Pharmacy Practice, Faculty of Pharmacy and Health Sciences, University of Baluchistan, Quetta, Pakistan; ^4^College of Medical Sciences, Azal University for Human Development, Sana'a, Yemen; ^5^Department of Clinical Pharmacy, University of Science and Technology of Fujairah, Fujairah, United Arab Emirates

**Keywords:** programmatic management of drug-resistant TB, PMDT: national tuberculosis control program, pharmacovigilance, antimicrobial resistance, adverse event (AE)

## Abstract

**Background:**

Treatment of multi-drug resistant tuberculosis (MDR-TB) for a prolonged period with comparatively less effective and more toxic second-line anti-TB drugs is associated with greater incidence of adverse events.

**Study aim:**

This study aimed to evaluate the frequency and factors associated with occurrence of adverse events among patients with MDR-TB attending the Bahawal Victoria Hospital, Bahawalpur, Pakistan.

**Study design:**

This retrospective study included all patients with MDR-TB who were registered and treated at the study site between June 2014 and December 2016 and had their treatment outcomes available at the time of data collection (i.e., November 2018).

**Measures and outcomes:**

The Electronic Nominal Record System (ERNS) records, medical charts of patients, and laboratory reports were reviewed to obtain the data. Adverse events were reported as per the standard criteria recommended by the WHO. Multivariate binary logistic regression was used to find the independent factors associated with the occurrence of adverse events.

**Results:**

A total of 179 patients with MDR-TB were included in the final analysis. Out of these, 114 (63.7%) patients experienced at least one adverse event during the course of their treatment. Depression was the most common adverse events (33%), followed by nausea and vomiting (27.4%) and arthralgia (27.4%). The factors associated with the occurrence of adverse events included presence of comorbidity (adjusted odds ratio [*AOR*] 2.951; 95% *CI* 1.423, 6.118) and being employed (*AOR* 3.445; 95% *CI* 1.188, 9.993).

**Conclusion:**

Adverse events were prevalent in this cohort, however, resolved with the effective management approaches. Patients with identified factors for occurrence of adverse events need special attention and enhanced clinical management.

## Introduction

Multi-drug resistant tuberculosis (MDR-TB) is a type of TB caused by *Mycobacterium tuberculosis* strain resistant to at least two most powerful anti-TB drugs; isoniazid and rifampicin (1). Globally, in 2018, 10 million new cases of TB were reported, while the incidence of rifampicin-resistant TB (RR-TB) was approximately five million (417,000–556,000) of which about 78% cases had MDR-TB (2). Various factors are associated with the development of MDR-TB, such as unsupervised treatment, delays in the initiation of treatment, inappropriate drug regimens, availability of anti-TB drugs and other antibiotics without prescription, poor quality of drugs provided, human errors, genetic factors, and most importantly patient non-adherence (3–5).

In addition to being a country with high consumption and irrational use of antibiotics (6–9), Pakistan is among the top countries where treatment options for many infectious diseases have become ineffective due to development of resistance against causative agents (10–17). Pakistan ranks fourth in the world among the high burden MDR-TB countries, while ranks first in the Eastern Mediterranean Region (2). According to the available evidence, a surge in the estimated number of MDR-TB/RR-TB cases has been seen over the past few years (i.e., increased from 26,000 cases in 2015 to 28,000 cases in 2018) (18, 19). The patients with drug resistant TB (DR-TB) (both RR-TB and MDR-TB) are treated at the Programmatic Management of Drug-resistant TB (PMDT) units of the National TB Control Program (NTP) of Pakistan that provide standardized and individualized care to the patients and closely monitor them to achieve mission statement of NTP, “a TB free Pakistan” (20).

The minimum treatment duration of MDR-TB is 18 months post sputum culture conversion (SCC) with the second line anti-TB drugs (SLDs); however, it may last up to 24 months (21, 22). Unlike DS-TB, second line therapy for MDR-TB is less effective, complex, toxic, and expensive. Moreover, compared with first line anti-TB drugs (FLDs) used for the treatment of DS-TB, a greater fraction of patients with MDR-TB experiences adverse events with SLDs that may range from minor illness to life threatening complications. The occurrence of adverse events not only compromise the health of the patients in terms of morbidity, mortality, and quality of life, but also negatively influence the healthcare systems, such as increase in the cost of treatment and length of hospitalizations (23–25). In this context, the WHO and NTP (1, 26) have recommended an early identification and management of adverse events among the patients with MDR-TB. The management strategies for adverse events vary based on needs of the patient which may range from non-pharmacological interventions (e.g., counseling) to pharmacological interventions (e.g., addition of symptomatic treatment, dose reduction, or temporary discontinuation of offending drug) (27–29).

With regard to Pakistan, little is known about the frequency of adverse events, and to date, a few studies from the country have reported the occurrence of adverse events among patients with MDR-TB (30, 31). However, no study from PMDT at the Bahawal Victoria Hospital (BVH) site has evaluated the occurrence of adverse events among patients with MDR-TB. Therefore, the aim of this study was to investigate the type, frequency, and factors associated with occurrence of adverse events among patients with MDR-TB treated at PMDT unit of the BVH, Bahawalpur, Pakistan.

## Methodology

### Study Setting

This study was conducted at the PMDT site of the NTP of Pakistan established at the Chest Diseases Unit (CDU) of the BVH, Bahawalpur, Pakistan. The CDU provides free of cost care and medicines to the patients with DS-TB and DR-TB under the supervision of doctors, pharmacists, treatment coordinators, and other supportive members (21, 32). A fully equipped laboratory is established at the MDR-TB section for TB-related investigations, such as smear microscopy and Xpert-MTB/RIF. For drug susceptibility testing (DST), specimen samples are sent to the National Reference Laboratory (NRL) located in Islamabad, Pakistan. In addition, the radiology and pathology departments of the BVH provide services to patients with MDR-TB on daily basis.

### Study Design

This was a retrospective record review (33) of all the confirmed patients with MDR-TB diagnosed and enrolled at the study site from June 2014 to December 2016. All patients with MDR-TB for whom the final treatment outcome available at the time of data collection were included in the study. The patients with mono, poly, and extensively drug-resistant TB (XDR-TB) were excluded from the study (4, 21). In addition, the patients with extra-pulmonary MDR-TB were excluded because of expected differences in an adverse event profile due to the involvement of other organ(s) and response of body to the treatment. Besides, patients having unknown or undefined drug resistance patterns were not included in the analysis.

### Data Collection

The data were collected in November 2018. The Electronic Nominal Record System (ENRS) records, medical charts of patients, and laboratory reports were reviewed to obtain socio-demographic, clinical, and treatment related data. The socio-demographic data included information of patient on age, gender, education, marital status, occupation, smoking status, residence, and household size. The clinical data included registration group, presence of comorbidity, baseline body weight, cavity disease, TB specific symptoms, family history of TB, history of streptomycin, and SLD use. The treatment related data contained information related to the treatment regimens given to the patients and any further removal or addition of drugs from the regimen. The adverse events related data included type and severity of adverse events, and its management.

### Treatment Protocol and Adverse Events Detection and Management

All presumptive patients with MDR-TB were referred to this PMDT site from the DS-TB section of the BVH and from other healthcare centers within Bahawalpur division. The presumptive patients with MDR-TB were initially screened for *M. tuberculosis* and rifampicin resistance by using smear microscopy and Xpert-MTB/RIF, respectively (21). Patients with positive results of smear microscopy and rifampicin resistance were started with empiric therapy comprising of one injectable aminoglycoside (i.e., amikacin/kanamycin/capreomycin) + levofloxacin + ethionamide + cycloserine + pyrazinamide + vitamin B6 (plus para amino salicylic acid for patients with documented history of MDR-TB treatment or previous use of SLDs) (21, 26). Meanwhile, sputum specimens of the patients were sent to the NRL for culture and DST against FLDs and SLDs. Depending upon the availability of DST results, patients were switched to tailored individualized regimen comprised of at least four effective SLDs (for which DST has confirmed susceptibility) or likely effective drugs (for which DST results were not available but had not been taken by patient for more than 1 month). Patient were treated for at least 18 months post SCC defined as “two consecutive negative sputum cultures taken at least 30 days apart following a positive sputum culture” (34). An injectable SLD was administered for a minimum of 8 months with 6 months of post SCC. Baseline and follow-up laboratory investigations included, complete blood count (CBC) with erythrocyte sedimentation rate, liver function tests (LFTs), renal function tests (RFTs), urine analysis, random blood glucose (RBG), serum uric, serum electrolytes, and screening for hepatitis C and B and HIV. In some patients, physicians recommended audiometry and visual tests. All patients were treated as out-patients and evaluated monthly. Treatment adherence at homes was ensured by trained treatment supporters and visits of Home DOTS Linkage (HDL) facilitator.

The adverse events were identified, documented, and managed as per criteria set by the NTP for PMDT sites (26). At the start of MDR-TB therapy, the patients were screened for any pre-existing symptoms. Afterward, all patients were evaluated by a clinician and psychologist (on monthly basis if not self-reported by the patient) for occurrence of adverse events. The adverse events were identified based on the judgment of clinician, laboratory confirmation, and/or self-reported by the symptomatic patients. The identified adverse events were documented in a standard adverse events reporting form and managed (i.e., symptomatic management with or without dose reduction of offending drug or removal of the offending drug) based on the national guidelines for PMDT. For adverse events which were confirmed by the laboratory testing, at least one abnormal value was considered sufficient to define the event (30, 31). Table 1 presents a detailed description of how the adverse events are identified.

**Table 1:** Identification of adverse drug events.

**Adverse drug events**	**Definition**
**Gastrointestinal disturbance**	
Nausea and vomiting	As reported by patient and documented by a clinician
Diarrhea	As reported by patient and reported by patent and documented by a clinician
Abdominal pain	As reported by patient and documented by a clinician
Gastritis	As reported by patient and documented by a clinician
Hepatotoxicity	One value of either transaminases or bilirubin at least five times higher than the upper normal limit without symptoms, or three times elevation of serum transaminases in the presence of symptoms
Anorexia	As reported by patient documented by a clinician
**Central nervous system disturbances**	
Dizziness and vertigo	As reported by patient and documented by a clinician
Headache	As reported by patient and documented by a clinician
Sleep disturbances (insomnia)	As reported by patient and documented by a clinician
Seizures	As documented by a psychologist
Paresthesia	Symptoms with burning and tingling of skin diagnosed by a clinician
**Psychiatric disturbances**	
Depression	As diagnosed by clinician or psychologist
Psychosis	As diagnosed by clinician or psychologist
**Ototoxicity**	
Hearing disturbance	As confirmed by audiometry or physical examination
Tinnitus	Persistent ringing in ears based on patient report
**Others**	
Nephrotoxicity	At least 1 serum creatinine value >130 umol/L^*^
Visual impairment	Difficulty in vision based on patient report
Arthralgia	Pain in joints as reported by patient and documented by clinician with or without presence of arthritis and elevation of uric acid >9 mg/dl^*^
Swelling	As documented by a clinician
Peripheral neuropathy	Pain, burning and/or numbness of extremities as diagnosed by a clinician or by electromyography
Allergic reaction	As documented by a clinician
Rash and pruritus	Signs of rash or dermatological reaction related to medicine diagnosed by a clinician
Weakness and fatigue	As reported by patient and documented by a clinician
Fever	As documented by a clinician
Dyspnea	Diagnosed by pulmonologist

* *Normal ranges: Alanine aminotransferase = 0–41 U/L; total bilirubin = up to 1 mg/dl; serum creatinine = 61.9–115 umol/L; uric acid = 2.5–8 mg/dl*.

### Data Analysis

Statistical analysis was conducted by using SPSS (Statistical Package for the Social Sciences, version 20, IBM Corp., NY, USA) for Windows^TM^. Continuous variables were presented as mean and SD, while categorical variables were presented as frequencies and percentages (%). Simple logistic regression analysis was used to find the association between the patient characteristics and occurrence of adverse events (dependent variable). The variables which were statistically significant in univariate analysis (i.e., *p* < 0.05) were entered into multivariate binary logistic regression analysis to find the factors independently associated with the occurrence of adverse events. A *p*-value of < 0.05 was set to be statistically significant (35). We would like to declare that this article used the same raw data on the basis of which an article has already been published (36). However, the present paper is entirely different in terms of its aim and objectives, and results.

## Results

### Description of the Patients

At the time of data collection, 397 patients with DR-TB were registered at study site. Among these, 204 patients were still on treatment. Upon receiving the DST results, one patient was mono-drug resistant, three were poly drug resistant, and eight were patients with XDR-TB. For two patients, drug resistance pattern was not defined. Therefore, 218 patients were excluded from the study. The remaining 179 patients, who had their final treatment outcomes at the time of data collection, were included in the study.

### Patients' Characteristics and Occurrence of Adverse Events

All included patients had pulmonary TB. There were almost equal proportion of male (*n* = 95, 53.1%) and female (*n* = 84, 46.9%) patients. The mean age of the patients was 37.5 (SD = 17.4) years. The majority of the patients (*n* = 114, 63.7%) were non-smokers. The mean baseline body weight of the patients was 42.7 (SD = 11.5) kg. More than 60% of the patients (*n* = 114, 63.7%) had comorbidities. The average number of TB-related symptoms were 4.5± 1.5 (median = 5), whereas the average number of comorbidities was 1.3 ± 0.5 (median = 1). Table 2 provides detailed information on socio-demographic and baseline clinical characteristics of the patients. Details on laboratory values at baseline, end of intensive phase, and end of treatment is provided in Supplementary File 1.

**Table 2:** Patients' characteristics and occurrence of adverse events (*n* = 179).

**Characteristics**	***n*** **(%)**	**Adverse event** ***n*** **(%)**	
		**Yes (*n* = 114)**	**No (*n* = 65)**
**Gender**			
Male	95 (53.1)	67 (70.5)	28 (29.5)
Female	84 (46.9)	47 (56)	37 (44)
**Age (years) (mean ±SD = 37.5 ±17.4)**			
5–14	11 (6.1)	6 (54.5)	5 (45.5)
15–24	41 (22.9)	25 (61)	16 (39)
25–34	36 (20.1)	22 (61.1)	14 (38.9)
35–44	31 (17.3)	23 (74.2)	8 (25.8)
45–54	20 (11.2)	14 (70)	6 (30)
55–64	23 (12.8)	17 (73.9)	6 (26.1)
>65	17 (9.5)	7 (41.2)	10 (58.8)
**Marital status**			
Unmarried	46 (25.7)	30 (65.2)	16 (34.8)
Married	87 (48.6)	66 (75.9)	21 (24.1)
Widow/divorced	8 (4.5)	3 (37.5)	5 (62.5)
Missing^*^	38 (21.2)	–	–
**Residence**			
Rural	144 (80.4)	91 (63.2)	53 (36.8)
Urban	35 (19.6)	23 (65.7)	12 (34.3)
**Occupation**			
Unemployed	87 (48.6)	45 (51.7)	42 (48.3)
Public employee	7 (3.9)	5 (71.4)	2 (28.6)
Private employee	54 (30.2)	43 (79.6)	11 (20.4)
Self employed	25 (14)	17 (68)	8 (32)
Missing^*^	6 (3.4)	–	–
**Education**			
No formal education	78 (43.6)	57 (73.1)	21 (26.9)
Primary levels	33 (18.4)	21 (63.6)	12 (36.4)
Secondary levels	11 (6.1)	8 (72.7)	3 (27.3)
University levels	6 (3.4)	5 (83.3)	1 (16.7)
Missing^*^	51 (28.5)	–	–
**Household size**			
<7	106 (59.2)	70 (66)	36 (34)
≥7	56 (31.3)	38 (67.9)	18 (32.1)
Missing^*^	17 (9.5)		
**Smoking status**			
Non smoker	114 (63.7)	65 (57)	49 (43)
Active smoker	33 (18.4)	26 (78.8)	7 (21.2)
Ex-smoker	22 (12.3)	18 (81.8)	4 (18.2)
Missing^*^	10 (5.6)	–	–
**Registration type**			
New	8 (4.5)	6 (75)	2 (25)
Previously treated	130 (72.6)	84 (64.6)	46 (35.4)
Relapse	3 (1.7)	2 (66.7)	1 (33.3)
Treatment after failure	28 (15.6)	17 (60.7)	11 (39.3)
Treatment after lost to follow up	7 (3.9)	3 (42.9)	4 (57.1)
Unknown previous history	3 (1.7)	2 (66.7)	1 (33.3)
**Baseline body weight (kg) (mean ±SD = 42.7 ±11.5)**			
≤ 40	89 (49.7)	49 (27.4)	40 (22.3)
>40	90 (50.3)	65 (36.3)	25 (14)
**Lung cavitation at baseline**			
Yes	130 (72.6)	89 (68.5)	41 (31.5)
No	1 (0.6)	1 (100)	0 (0)
Missing^*^	48 (26.8)	–	–
**TB specific symptoms**			
Cough	120 (67)	87 (72.5)	33 (27.5)
Fever	111 (62)	80 (72.1)	31 (27.9)
Breathlessness	81 (45.3)	61 (75.3)	20 (24.7)
Night sweating	55 (30.7)	45 (81.8)	10 (18.2)
Weight loss	86 (48)	63 (73.3)	23 (26.7)
Chest pain	78 (43.6)	58 (74.4)	20 (25.6)
Hemoptysis	23 (12.8)	16 (69.6)	7 (30.4)
**History of second-line drug use**			
Yes	18 (10.1)	10 (55.6)	8 (44.4)
No	161 (89.9)	104 (64.6)	57 (35.4)
**History of streptomycin use**			
Yes	52 (29.1)	33 (63.5)	19 (36.5)
No	127 (70.9)	81 (63.8)	46 (36.2)
**Family history of tuberculosis**			
Present	21 (11.7)	16 (76.2)	5 (23.8)
Absent	122 (68.2)	84 (68.9)	38 (31.1)
Missing^*^	36 (20.1)	–	–
**Comorbidity**			
Yes	114 (63.7)	86 (75.4)	28 (24.6)
No	63 (35.2)	27 (42.9)	36 (57.1)
Missing^*^	2 (1.1)	–	–
**Comorbidity type**			
Diabetes mellitus	23 (12.8)	17 (73.9)	6 (26.1)
Liver disease	5 (2.8)	4 (80)	1 (20)
Lung disease	100 (55.9)	76 (76)	24 (24)
Heart diseases	3 (1.7)	3 (100)	0 (0)

* *Data were not available inpatient medical records*.

### Treatment Regimen of the Patients

The majority of the patients were on four or more drugs throughout the course of their treatment. Initially, the empiric therapy was initiated in all patients (*n* = 179). On the receipt of DST results, treatment was modified in 13 (7.3%) patients. The most common drug added later to the regimen was levofloxacin (*n* = 8, 4.5%; Table 3).

**Table 3:** Treatment regimen of the patients (*n* = 179).

**Group**	**Drugs** **(abbreviation)**	**Drugs added initially to regimen** ***n* (%)**	**Drugs added later to regimen** ***n* (%)**	**Drugs removed from regimen** ***n* (%)**	**Standard doses**
**A: Fluoroquinolones**					
	Levofloxacin (Lfx)	151 (84.4)	8 (4.5)	2 (1.1)	7.5–10 mg/kg (max. dose 1,000 mg)
	Moxifloxacin(Mfx)	28 (15.6)	0 (0)	0 (0)	400 mg once daily
**B: Second-line injectable**					
	Amikacin(Am)	157 (87.7)	1 (0.6)	129 (72.1)	15–20 mg/kg (max. dose 1,000 mg) 6 d/wk
	Capreomycin(Cm)	22 (12.3)	0 (0)	0 (0)	15–20 mg/kg (max. dose 1,000 mg) 6 d/wk
**C: Other core second line agents**					
	Ethionamide(Eto)	176 (98.3)	0 (0)	0 (0)	15–20 mg/kg (max. dose 1,000 mg)
	Prothionamide(Pto)	0 (0)	0 (0)	0 (0)	15–20 mg/kg (max. dose 1,000 mg)
	Linezolid (Lzd)	17 (9.5)	1 (0.6)	0 (0)	600 mg once daily
	Cycloserine(Cs)	179 (100)	0 (0)	0 (0)	15–20 mg/kg (max. dose 1,000 mg)
	Clofazimine(Cfz)	10 (5.6)	0 (0)	0 (0)	100 mg once daily
**D: Add-on agents**					
**D1**					
	Ethambutol(E)	52 (29.1)	2 (1.1)	0 (0)	25 mg/kg daily (max. dose 2,000 mg)
	Pyrazinamide (Z)	177 (98.9)	0 (0)	0 (0)	30–40 mg/kg daily (max. dose 2,500 mg)
	High dose isoniazid (H-Inh)	6 (3.4)	0 (0)	0 (0)	16–20 mg/kg once daily (max. dose 1,500 mg)
**D2**					
	Bedaquiline(Bdq)	0 (0)	0 (0)	0 (0)	–
	Delamanid(Dlm)	0 (0)	0 (0)	0 (0)	–
**D3**					
	Amoxicillin-clavulanate(Amx-Clv)	15 (8.4)	0 (0)	0 (0)	1,500/375 mg daily
	Clarithromycin (Clr)	16 (8.9)	0 (0)	0 (0)	1,000 mg daily
	Paraamino salicylic acid (PAS)	63 (35.2)	1 (0.6)	0 (0)	150 mg/kg (max. dose 12 gm)

*mg, milligram; kg, kilogram; d/wk, days per week*.

### Drug Resistance Pattern in the Patients

About 60% of the patients (*n* = 103, 57.5%) were resistant to both isoniazid and rifampicin, while 5% (*n* = 9) were resistant to all five FLDs. Similarly, 29% of the patients (*n* = 52) were resistant to ofloxacin. A detailed description of drug resistance pattern is presented in Supplementary File 2.

### Types of Adverse Events and the Management Strategy

Among 179 (100%) patients, at least one adverse event was experienced by 114 (63.7%) patients. An average of 2.4 ± 1.5 (median = 2) adverse events were experienced by the patients. Depression (*n* = 59, 33%) was most reported adverse event followed by nausea and vomiting (*n* = 49, 27.4%), arthralgia (*n* = 49, 27.4%), hearing disturbance (*n* = 16, 8.9%), psychosis (*n* = 13, 7.3%), dyspnea (*n* = 12, 6.7%), and anorexia (*n* = 11, 6.1%). Similarly, peripheral neuropathy was reported in 9 (5%) patients, headache in 7 (3.9%) patients, dizziness and vertigo in 7 (3.9%) patients, gastritis in 6 (3.4%) patients, and rash and pruritus in 6 (3.4%) patients. Life threatening adverse events, such as nephrotoxicity were rare in this cohort (*n* = 5, 2.8%). The management of adverse events ranged from non-pharmacological interventions (i.e., counseling) to pharmacological interventions [i.e., dose reduction, addition of new drug (supportive therapy), counseling, and temporary discontinuation of causative drug]. In most of the cases, the adverse events resolved with one of the strategies mentioned in Table 4. A detailed description on the potential causative drug of each adverse event, their severity and seriousness, strategies adopted to resolve adverse events, and the outcome of these resolution strategies is presented in Supplementary File 3.

**Table 4:** Type of adverse events recorded and the management strategy.

**Adverse events**	**Frequency** ***n* (%)**	Potential culprit drugs ^*^^†^	**Interventions in MDR-TB regimen^‡^** ***n* (%)**	**Type of intervention**				**Outcome of modification** 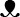	
				**Dose reduction** ***n* (%)**	**New drug added** ***n* (%)**	**Counseling** ***n* (%)**	**Temporary discontinuation** ***n* (%)**	**Resolved** ***n* (%)**	**Not resolved** ***n* (%)**
Depression	59 (33)	Cs (59)	57 (31.8)	–	54 (30.2)	1 (0.6)	2 (1.1)	56 (31.3)	2 (1.1)
Nausea and vomiting	49 (27.4)	Z (34), Lfx (1), Eto (4), PAS (4)	48 (26.9)	8 (4.5)	34 (19)	3 (1.7)	3 (1.7)	43 (24)	4 (2.2)
Arthralgia	49 (27.4)	Z (47), Lfx (1)	48 (26.9)	3 (1.7)	43 (24)	–	2 (1.1)	46 (25.7)	3 (1.7)
Hearing disturbance	16 (8.9)	Am (15), Cs (1)	16 (8.9)	10 (5.6)	–	–	6 (3.4)	13 (7.3)	2 (1.1)
Psychosis	13 (7.3)	Cs (13)	13 (7.3)	1 (0.6)	8 (4.5)	–	4 (2.2)	7 (3.9)	5 (2.8)
Dyspnea	12 (6.7)	Z (2), PAS (1)	9 (5)**^ϕ^**	–	9 (5)	–	–	9 (5)	–
Anorexia	11 (6.1)	Z (2), Lfx (1), Eto (1), PAS (1)	8 (4.5)**^ϕ^**	1 (0.6)	4 (2.2)	3 (1.7)	–	8 (4.5)	2 (1.1)
Peripheral neuropathy	9 (5)	Z (2), Lfx (1), Cs (5), PAS (1)	9 (5)	1 (0.6)	5 (2.8)	–	3 (1.7)	8 (4.5)	1 (0.6)
Headache	7 (3.9)	Z (5), PAS (1)	6 (3.4)**^ϕ^**	–	6 (3.4)	–	–	7 (3.9)	–
Dizziness and vertigo	7 (3.9)	Z (2), Lfx (1), Cs (2)	7 (3.9)	–	6 (3.4)	1 (0.6)	–	6 (3.4)	1 (0.6)
Gastritis	6 (3.4)	Z (6)	6 (3.4)	–	4 (2.2)	–	2 (1.1)	6 (3.4)	–
Rash and pruritus	6 (3.4)	Z (2), Am (1)	5 (2.8)	1 (0.6)	3 (1.7)	–	1 (0.6)	5 (2.8)	–
Visual impairment	5 (2.8)	E (4)	5 (2.8)	1 (0.6)	1 (0.6)	–	3 (1.7)	5 (2.8)	–
Weakness and fatigue	5 (2.8)	PAS (1)	2 (1.1)**^ϕ^**	–	1 (0.6)	1 (0.6)	–	4 (2.2)	–
Nephrotoxicity	5 (2.8)	Am (1), Z (2), Cm (1), Eto (1)	5 (2.8)	1 (0.6)	–	–	4 (2.2)	4 (2.2)	1 (0.6)
Abdominal pain	5 (2.8)	Z (1), PAS (1)	4 (2.2)	1 (0.6)	1 (0.6)	1 (0.6)	1 (0.6)	4 (2.2)	–
Allergic reactions	4 (2.2)	Z (2), Am (2)	4 (2.2)	1 (0.6)	1 (0.6)	–	2 (1.1)	4 (2.2)	–
Fever	4 (2.2)	Cs (1), Lfx (1)	4 (2.2)	–	4 (2.2)	–	–	3 (1.7)	–
Diarrhea	3 (1.7)	Z (1), PAS (1)	3 (1.7)	2 (1.1)	1 (0.6)	–	–	3 (1.7)	–
Seizures	2 (1.1)	Cs (2)	2 (1.1)	–	1 (0.6)	–	1 (0.6)	2 (1.1)	–
Tinnitus	2 (1.1)	Am (1), Eto (1)	2 (1.1)	1 (0.6)	-	–	1 (0.6)	2 (1.1)	–
Sleep disturbance	1 (0.6)	Cs (1)	1 (0.6)	–	1 (0.6)	–	–	1 (0.6)	–
Paresthesia	1 (0.6)	Cs (1)	1 (0.6)	–	1 (0.6)	–	–	–	–
Swelling	1 (0.6)	Z (1)	1 (0.6)	–	1 (0.6)	–	–	1 (0.6)	–

* *Abbreviation of drugs used that were reported to cause adverse drug reactions in patients (Cs, cycloserine; Z, pyrazinamide; Lfx, levofloxacin; Eto, ethionamide; PAS, para amino salicylic acid; Am, amikacin; E, ethambutol)*.

† *Data related to drugs implicated were not available in the records of patients (n = 6 nausea and vomiting, n = 1 arthralgia, n = 9 dyspnea, n = 6 anorexia, n = 1 headache, n = 2 dizziness and vertigo, n = 3 rash and pruritus, n = 1 visual impairment, n = 4 weakness and fatigue, n = 3 abdominal pain, n = 2 fever, n = 1 diarrhea)*.

‡ *Data related to modification in multi-drug resistant TB regimen were not available in the records of patients (n = 2 depression, n = 1 nausea and vomiting, n = 1 arthralgia, n = 2 dyspnea, n = 2 anorexia, n = 1 rash and pruritus, n = 2 weakness and fatigue, n = 1 abdominal pain)*.

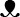
*Outcome of modification were reported for whom the data were available in ENRS records*.

Φ *No action was taken in patients (n = 1 dyspnea, n = 1 anorexia, n = 1 headache, n = 1 weakness and fatigue)*.

### Factors Associated With Occurrence of Adverse Events

In simple logistic regression, the variables that were significantly associated with the occurrence of adverse events included; male gender (odds ratio [*OR*] 1.884; 95% *CI* 1.017, 3.490; *p*-value 0.044), being employed (*OR* 2.889; 95% *CI* 1.512, 5.518; *p*-value 0.001), being a smoker (*OR* 3.015; 95% *CI* 1.414, 6.433; *p*-value 0.004), having ≤ 40 kg baseline body weight (*OR* 0.471; 95% *CI* 0.253, 0.878; *p*-value 0.018), and having a comorbidity (*OR* 4.095; 95% *CI* 2.124, 7.895; *p*-value < 0.000). In multivariate binary logistic regression analysis, the factors which remained statistically associated with the occurrence of adverse events included were being employed (adjusted *OR* [*AOR*] 3.445; 95% *CI* 1.188, 9.993; *p*-value 0.023) and having a comorbidity (*AOR* 2.951; 95% *CI* 1.423, 6.118; *p*-value 0.004) (Table 5).

**Table 5:** Factors associated with the occurrence of adverse events.

**Variable**	**Adverse event**		**Univariate**		**Multivariate**	
	**Yes** ***n* = 114**	**No** ***n* = 65**	***p-*** **value**	**OR (95% CI)**	***p-*** **value**	**AOR (95% CI)**
**Gender**						
Female	47	37		Referent		Referent
Male	67	28	**0.044**	1.884 (1.017, 3.490)	0.230	0.477 (0.143, 1.596)
**Employed**						
No	45	42		Referent		Referent
Yes	65	21	**0.001**	2.889 (1.512, 5.518)	**0.023**	3.445 (1.188, 9.993)
**Smoking status**						
Non-smoker	65	49		Referent		Referent
Smoker + ex-smoker	44	11	**0.004**	3.015 (1.414, 6.433)	0.372	1.627 (0.559, 4.739)
**Comorbidity**						
No	27	36		Referent		Referent
Yes	86	28	**<0.0005**	4.095 (2.124, 7.895)	**0.004**	2.951 (1.423, 6.118)
**Baseline weight (kg)**						
>40	65	25		Referent		Referent
≤ 40	49	40	**0.018**	0.471 (0.253, 0.878)	0.375	0.701 (0.320, 1.537)

*p < 0.05 significant in bold. Model summary: chi-square (25.328), df (5), p < 0.01; Nagelkerke R^2^ (0.200); Hosmer & Lameshow chi-square test (5.164), df (8), p = 0.7*.

## Discussion

The treatment of MDR-TB is a global challenge mainly due to the complexity of second line drug therapy. In this study, we found that about 64% of patients with MDR-TB experienced adverse events. However, most of adverse events resolved with the interventions of clinicians and none of them lead to permanent discontinuation of any of SLD.

The frequency of adverse events in our study were consistent with that reported in studies from Turkey (69%) (37), India (58%) (33), and Russia (73.3%) (38). However, high incidence rate was documented by studies conducted in Latvia (79%) (39), Italy (89.2%) (40), and China (90.7%) (22). Similarly, two studies from Pakistan reported that the frequency of adverse events among patients with MDR-TB ranged from 72.3 to 77% (30, 31). In contrast, low incidence rate of adverse events was reported in studies conducted in India (46.9%) (23), Ethiopia (51%) (41), and Nigeria (44%) (42). The difference in the incidence rate of adverse events among studies might be attributable to differences in health system and patient related factors. For example, differences in the definition of adverse events and ability of clinicians to detect adverse events, attitude and practices of healthcare workers regarding adverse events reporting, pattern of drug use and variation in the dosage regimen of anti-TB drugs, and timely administration of ancillary drugs to mitigate the effect of adverse events (22, 23, 27, 30, 31, 43). Patient related factors include their nutritional status, ethnicity, age, their perception about the disease and its therapeutic regimen, and presence of comorbidity (22, 41).With regard to the perception about the disease and its therapeutic regimen as a predictor of adverse events, a study from the same site explored views of patients and reported that they were not provided with adequate information about the disease and treatment plan (44). Poor knowledge leads to patient's consideration of the common side effects of TB medicines as harmful and dangerous, thereby increasing report of adverse events (45). Regarding the presence of comorbidity, a plethora of published studies (28, 41, 46) has reported that patients with any type of comorbid conditions were at increased risks of experiencing adverse drug events. This is probably owing to the polypharmacy and subsequent risk of drug-drug interactions, compromised immunity due to additional medical conditions, and poor tolerance to drugs (41, 47).

In our study, psychiatric illness (depression = 33% and psychosis = 7.3%) was the most significant adverse events documented in most of the patients. This rate coincides with an earlier Pakistani study (29.3%) (30) and a study from Egypt (26.5%) (48). However, studies from other countries reported psychiatric illness rate ranging from 6.2 to 21.3% (28, 37, 49–51). In addition to the central nervous system effects of cycloserine, ethionamide, and fluoroquinolones, the relatively high frequency of psychiatric disturbance in current cohort could be attributed to many other factors. An overwhelming majority (93.5%) of present study participants had a documented history of previous episodes of TB treatment. The adverse outcomes, fear, and fatigue of previous unsuccessful episodes of TB treatment might have contributed to the deterioration of mental health of participants. Poor socio-economic status, chronic nature of the MDR-TB and its prolonged therapy, the endocrine reactions resulting from overproduction of interleukin-6 in chronic bacterial infections, and lack of motivation and communication of patient with the healthcare team could be the other possible factors of high frequency of poor mental health in these patients (27). According to the WHO, cycloserine, ethionamide, and fluoroquinolones are the drugs which are most likely to cause psychiatric disturbances (52). However, at our study site, it was only attributed to cycloserine. Psychiatric disturbance is one of the most problematic issue that may require permanent discontinuation of causative drugs because of the development of suicidal tendency. Studies from Pakistan and India reported that cycloserine was discontinued in few patients due to depression and psychosis (23, 30, 33). In current cohort, the psychiatric disturbance did not lead to permanent discontinuation any SLD, however, cycloserine was temporarily discontinued in six patients. Majority of patients with psychiatric disturbance have successfully managed with counseling and prescription of anti-depressants. With this strategy, depression was resolved in 56 out of 59 cases, while psychosis was resolved in seven out of 13 cases. A recent meta-analysis concluded that cycloserine could be removed from the empiric therapy of patient without compromising the treatment success. The meta-analysis further recommended to use fluoroquinolones, clofazimine, and bedaquiline (depending upon availability) unless otherwise specified by DST results as these were reported to cause less adverse drug reactions (53).

In this study, comparatively lower percentage of patients reported various gastrointestinal (GI) adverse events [i.e., nausea and vomiting (27.4%), anorexia (6.1%), gastritis (3.4%), and abdominal pain (2.8%)]. The incidence of nausea and vomiting was consistent with the two Indian (20%) and one Pakistani (34%) studies (23, 30, 33). Higher incidence of GI related adverse events were reported in studies conducted in Ethiopia (59%) (54), India (71%) (27), and Russia (75%) (38). The subjective nature of most of GI symptoms could be one of the possible reasons for widespread variation in the reported frequency of these symptoms in different studies. Among 49 patients having nausea and vomiting, the dose of offending drug was reduced in eight patients, while it was temporarily discontinued in three patients. Most of the patients (*n* = 34) received symptomatic remedy (i.e., antiemetic) to resolve the problem, and this treatment strategy worked for them. Similarly, other GI symptoms were resolved by adding ancillary drugs. Additionally, a Pakistani study by Ahmad and co-worker reported that most of GI related adverse events were resolved through palliative care and counseling (30).

Besides, the patients in this study complained of musculoskeletal pain (arthralgia in 27.4%) after the administration of SLDs which is close to that reported from Ethiopia (34%) (54) and Namibia (26%) (55). In contrast, studies from China (56.4%) (22) and Russia (47.1%) (38) reported higher frequency of arthralgia in patients with MDR-TB. While, studies from India (14%) (27) and Peru (6.7%) (28) reported lower incidence of arthralgia among patients. Interestingly, in Pakistani studies, occurrence of arthralgia ranged from 24 to 48% (30, 31). Variability in the findings among studies may be due to the subjective nature of the adverse event. In published literature, pyrazinamide is one of the frequently reported culprit of arthralgia in patients with MDR-TB. However, in current study, the musculoskeletal pain subsided in most of the patients without permanent discontinuation of pyrazinamide, and this is in line with the findings of a Pakistani study (30).

A smaller proportion of patients (*n* = 16, 8.9%) experienced hearing disturbance with anti-TB therapy. Other studies from Pakistan (21%) (30), Lativa (19%) (39), Russia (14.6%) (38), Namibia (25%) (51), and Turkey (41.8%) (37) reported higher incidence of hearing disturbances among patients with MDR-TB. Lack of audiometry at regular intervals could be the possible reasons of lower incidence of ototoxicity in current cohort. This is alarming because if such patients are left unintended, it may lead to hearing loss. In current cohort, the ototoxicity was managed by dose reduction and temporary discontinuation of amikacin in 10 and 5 patients, respectively. Ototoxicity completely disappeared in 13 (out of 16) patients when amikacin therapy was discontinued in intensive phase of treatment.

Similar to other studies, life threatening nephrotoxicity was uncommon in our study cohort, and it resolved in four (out of five) patients with the temporary discontinuation and dose reduction of offending drugs (28, 30, 38, 54). Some other adverse events that were rare in this cohort were dyspnea, peripheral neuropathy, headache, dizziness and vertigo, rash and pruritus, and visual impairment. Propitiously, no case of hypokalemia, hypothyroidism, hepatitis, liver injury, and dehydration was reported in our study.

Our study reported higher probability of developing adverse events among those patients who had comorbidities. This finding is supported by published studies elsewhere (28, 41, 46, 56, 57). One of the possible reasons of developing adverse events in this sub-group of patients might be due to polypharmacy and resultant drug–drug interactions (54, 58, 59). In addition, our study findings demonstrated that employed patients tend to experience more adverse events as compared with unemployed. Interestingly, no such association has been reported in the existing literature and warrants further exploration of the underlying reasons. One possible reason could be their active lifestyle and comparatively greater awareness about the drugs adverse effects making them more anxious and communicative of unwanted effects of drugs to the healthcare team.

Interestingly, the findings of our study showed that the patients who experienced adverse events associated with MDR-TB drugs had higher probability of successful treatment outcomes [multivariate analysis to find predictors of treatment success is beyond the scope of this paper, and is available elsewhere (36)]. The current finding might be due to the fact that patients experiencing adverse events were given careful and individualized care (36). As a result, not only their adverse events were resolved, but they were more likely to achieve successful outcomes. These findings point toward the need for enhanced provision of pharmacist-led patient-centered care for all patients, as pharmacists have effective consultation skills, as well as proven expertise related to the different aspects of medicine, such as management of adverse events following medication (32, 60–62).

This study has some limitations. First, due to retrospective nature of the study, some important characteristics of patients, for example, body mass index (BMI), drugs used for comorbid conditions, and concurrent use of complementary therapies were not recorded. These confounders can influence the incidence of adverse events. Second, reporting behavior for subjective adverse events (e.g., nausea, vomiting, anorexia, and headache) may vary among patients. Third, at the time of enrollment of the patients, audiometry was not performed. This might have underestimated ototoxicity. Based on these limitations, we recommend a large prospective multicenter study in future.

## Conclusion

In current study, adverse events were highly prevalent but were managed by supportive pharmacological and non-pharmacological interventions. Despite high prevalence, adverse events neither lead to temporary or permanent discontinuation of MDR-TB treatment nor adversely affected the final treatment outcomes. However, patients who experienced adverse events associated with MDR-TB drugs had higher probability of successful treatment outcomes, thereby highlighting focus on individualized care of all patients irrespective of occurrence of adverse events. The high incidence of depression in our study is alarming and can be managed by adopting the recently recommended cycloserine lacked treatment regimen for patients with MDR-TB. Regular audiometry of all patients and close monitoring and enhanced clinical management of patients with identified risk factors for adverse events are suggested.

## Data Availability Statement

The original contributions presented in the study are included in the article/Supplementary Material, further inquiries can be directed to the corresponding author/s.

## Ethics Statement

The studies involving human participants were reviewed and approved by the Pharmacy Research and Ethics Committee (PREC) at the Islamia University Bahawalpur, Pakistan (Reference number: 98/S-2018-/PREC). Written informed consent for participation was not required for this study.

## Author Contributions

MA: supervision and roles/writing-review and editing. MA, WaqA, and NA: conceptualization. MA, WaqA, WajA, IM, MI, YA-W, and NA: methodology. WaqA: writing-original draft. MA, WajA, IM, MI, YA-W, and WaqA: formal analysis. WajA, IM, MI, MA, NA, and YA-W: validation. NA: roles/writing-original draft. WajA, IM, MI, and YA-W: writing-review and editing. All authors have read and approved the manuscript.

## Conflict of Interest

The authors declare that the research was conducted in the absence of any commercial or financial relationships that could be construed as a potential conflict of interest.

## Publisher's Note

All claims expressed in this article are solely those of the authors and do not necessarily represent those of their affiliated organizations, or those of the publisher, the editors and the reviewers. Any product that may be evaluated in this article, or claim that may be made by its manufacturer, is not guaranteed or endorsed by the publisher.
