# Supplementary material for: Frequency and Factors Associated With Adverse Events Among Multi-Drug Resistant Tuberculosis Patients in Pakistan: A Retrospective Study
Source: Front Med (Lausanne). 2022 Mar 1;8:790718. doi: 10.3389/fmed.2021.790718 (PMC8922404; doi:10.3389/fmed.2021.790718)
Supplement: Supplementary file 1 [file Table_1.DOCX]

**Supplementary File 1**

Laboratory values of multi-drug resistant tuberculosis patients (N = 179)

| **Variable** | **Patient n (%)** | **Adverse event** | |
| --- | --- | --- | --- |
|  |  | **Yes n (%)** | **No n (%)** |
| **Baseline HB level** |  |  |  |
| Below Normal | 58 (32.4) | 39 (67.2) | 19 (32.8) |
| Normal | 17 (9.5) | 11 (64.7) | 6 (35.3) |
| Above Normal | 0 (0) | - | - |
| Missing | 104 (58.1) | - | - |
| **HB level at end of IP** |  |  |  |
| Below Normal | 39 (21.8) | 32 (82.1) | 7 (17.9) |
| Normal | 24 (13.4) | 22 (91.7) | 2 (8.3) |
| Above Normal | 0 (0) | - | - |
| Missing | 116 (64.8) | - | - |
| **HB level at end of CP** |  |  |  |
| Below Normal | 42 (23.5) | 35 (83.3) | 7 (16.7) |
| Normal | 21 (11.7) | 21 (100) | 0 (0) |
| Above Normal | 0 (0) | - | - |
| Missing | 116 (64.8) | - | - |
| **Baseline WBC level** |  |  |  |
| Below Normal | 1 (0.6) | 0 (0) | 1 (100) |
| Normal | 47 (26.3) | 32 (68.1) | 15 (31.9) |
| Above Normal | 19 (10.6) | 14 (73.7) | 5 (26.3) |
| Missing | 112 (62.6) | - | - |
| **WBC level at end of IP** |  |  |  |
| Below Normal | 0 (0) | - | - |
| Normal | 49 (27.4) | 42 (85.7) | 7 (14.3) |
| Above Normal | 11 (6.1) | 9 (81.8) | 2 (18.2) |
| Missing | 119 (66.5) | - | - |
| **WBC level at end of CP** |  |  |  |
| Below Normal | 0 (0) | - | - |
| Normal | 47 (26.3) | 40 (85.1) | 7 (14.9) |
| Above Normal | 15 (8.4) | 15 (100) | 0 (0) |
| Missing | 117 (65.4) | - | - |
| **Baseline SGOT level** |  |  |  |
| Below Normal | 0 (0) | - | - |
| Normal | 51 (28.5) | 33 (64.7) | 18 (35.3) |
| Above Normal | 2 (1.1) | 2 (100) | 0 (0) |
| Missing | 126 (70.4) | - | - |
| **SGOT level at end of IP** |  |  |  |
| Below Normal | 0 (0) | - | - |
| Normal | 30 (16.8) | 24 (80) | 6 (20) |
| Above Normal | 3 (1.7) | 2 (66.7) | 1 (33.3) |
| Missing | 146 (81.6) | - | - |
| **SGOT level at end of CP** |  |  |  |
| Below Normal | 0 (0) | - | - |
| Normal | 27 (15.1) | 23 (85.2) | 4 (14.8) |
| Above Normal | 2 (1.1) | 2 (100) | 0 (0) |
| Missing | 150 (83.8) | - | - |
| **Baseline SGPT level** |  |  |  |
| Below Normal | 0 (0) | - | - |
| Normal | 43 (24) | 28 (65.1) | 15 (34.9) |
| Above Normal | 3 (1.7) | 1 (33.3) | 2 (66.7) |
| Missing | 133 (74.3) | - | - |
| **SGPT level at end of IP** |  |  |  |
| Below Normal | 0 (0) | - | - |
| Normal | 32 (17.9) | 25 (78.1) | 7 (21.9) |
| Above Normal | 1 (0.6) | 1 (100) | 0 (0) |
| Missing | 146 (81.6) | - | - |
| **SGPT level at end of CP** |  |  |  |
| Below Normal | 0 (0) | - | - |
| Normal | 24 (13.4) | 19 (79.2) | 5 (20.8) |
| Above Normal | 2 (1.1) | 2 (100) | 0 (0) |
| Missing | 153 (85.5) | - | - |
| **Baseline Bilirubin level** |  |  |  |
| Below Normal | 0 (0) | - | - |
| Normal | 74 (41.3) | 52 (70.3) | 22 (29.7) |
| Above Normal | 3 (1.7) | 2 (66.7) | 1 (33.3) |
| Missing | 102 (57) | - | - |
| **Bilirubin level at end of IP** |  |  |  |
| Below Normal | 0 (0) | - | - |
| Normal | 64 (35.8) | 55 (85.9) | 9 (14.1) |
| Above Normal | 0 (0) | - | - |
| Missing | 115 (64.2) | - | - |
| **Bilirubin level at end of CP** |  |  |  |
| Below Normal | 2 (1.1) | 2 (100) | 0 (0) |
| Normal | 63 (35.2) | 56 (88.9) | 7 (11.1) |
| Above Normal | 0 (0) | - | - |
| Missing | 114 (63.7) | - | - |
| **Baseline Creatinine level** |  |  |  |
| Below Normal | 1 (0.6) | 1 (100) | 0 (0) |
| Normal | 71 (39.7) | 49 (69) | 22 (31) |
| Above Normal | 4 (2.2) | 3 (75) | 1 (25) |
| Missing | 103 (57.5) | - | - |
| **Creatinine level at end of IP** |  |  |  |
| Below Normal | 1 (0.6) | 1 (100) | 0 (0) |
| Normal | 64 (35.8) | 55 (85.9) | 9 (14.1) |
| Above Normal | 0 (0) | - | - |
| Missing | 114 (63.7) | - | - |
| **Creatinine level at end of CP** |  |  |  |
| Below Normal | 0 (0) | - | - |
| Normal | 65 (36.3) | 58 (89.2) | 7 (10.8) |
| Above Normal | 0 (0) | - | - |
| Missing | 114 (63.7) | - | - |
| **Baseline Glucose level** |  |  |  |
| Below Normal | 12 (6.7) | 9 (75) | 3 (25) |
| Normal | 8 (4.5) | 8 (100) | 0 (0) |
| Above Normal | 3 (1.7) | 3 (100) | 0 (0) |
| Missing | 156 (87.2) | - | - |
| **Glucose level at end of IP** |  |  |  |
| Below Normal | 9 (5) | 8 (88.9) | 1 (11.1) |
| Normal | 18 (10.1) | 16 (88.9) | 2 (11.1) |
| Above Normal | 4 (2.2) | 3 (75) | 1 (25) |
| Missing | 148 (82.7) | - | - |
| **Glucose level at end of CP** |  |  |  |
| Below Normal | 29 (16.2) | 27 (93.1) | 2 (6.9) |
| Normal | 11 (6.1) | 10 (90.9) | 1 (9.1) |
| Above Normal | 2 (1.1) | 2 (100) | 0 (0) |
| Missing | 137 (76.5) | - | - |

IP = Intensive Phase; CP = Continuous Phase; HB = Hemoglobin; SGPT = Serum Glutamate-Pyruvic Transaminase; SGOT = Serum Glutamic Oxaloacetic Transaminase; WBCs = White Blood Cells Normal ranges: HB = Male 14-18 g/dl, Female = 11-16 g/dl; WBCs = 3800 - 11,000/mm3; Creatinine = 0.2-1.5 mg/dl; SGOT = 0-35 U/L; SGPT = 0-35 U/L; Bilirubin = 0.2-1.4 mg/dl; Glucose = 65 – 110 mg/dl.
