# Supplementary material for: Frequency and Factors Associated With Adverse Events Among Multi-Drug Resistant Tuberculosis Patients in Pakistan: A Retrospective Study
Source: Front Med (Lausanne). 2022 Mar 1;8:790718. doi: 10.3389/fmed.2021.790718 (PMC8922404; doi:10.3389/fmed.2021.790718)
Supplement: Supplementary file 2 [file Table_2.DOCX]

Supplementary File 2

Drug resistance pattern in the patients

| **Variable** | **Patients**  **n (%)** | **Variable** | **Patients**  **n (%)** |
| --- | --- | --- | --- |
| **Resistance to FLDs** |  | **Resistance to SLDs** |  |
| **Name of drugs** |  | **Name of drugs** |  |
| Resistance to HR | 103 (57.5) | Resistance to ofloxacin | 52 (29.1) |
| Resistance to HRE | 14 (7.8) | Resistance to ethionamide | 1 (0.6) |
| Resistance to HRES | 22 (12.3) | Resistance to kanamycin | 2 (1.1) |
| Resistance to HRESZ | 9 (5) | Resistance to ofloxacin and pyrazinamide | 15 (8.4) |
| Resistance to HRS | 12 (6.7) | Resistance to ofloxacin and ethionamide | 1 (0.6) |
| Resistance to HRZ | 8 (4.5) | **Number of drugs** |  |
| Resistance to HRZS | 2 (1.1) | 0 | 126 (70.4) |
| Resistance to HREZ | 9 (5) | 1 | 51 (28.5) |
| **Number of drugs** |  | 2 | 2 (1.1) |
| 2 | 103 (57.5) |  |  |
| 3 | 34 (19) |  |  |
| 4 | 33 (18.5) |  |  |
| 5 | 9 (5) |  |  |

FLDs = first-line drugs, SLDs = second-line drugs, H = isoniazid, R = rifampicin, E = Ethambutol, S = streptomycin, Z = pyrazinamide
