# Supplementary material for: Frequency and Factors Associated With Adverse Events Among Multi-Drug Resistant Tuberculosis Patients in Pakistan: A Retrospective Study
Source: Front Med (Lausanne). 2022 Mar 1;8:790718. doi: 10.3389/fmed.2021.790718 (PMC8922404; doi:10.3389/fmed.2021.790718)
Supplement: Supplementary file 3 [file Table_3.DOCX]

Supplementary File 3

Detailed Summary of Top 12 Adverse events

1. **Depression**

| **Variable** | **n (%)** | |
| --- | --- | --- |
| **Incidence** | 59 (33) | |
| **Potential culprit drug** | | |
| Cycloserine | 59 (33) | |
| **Severity of ADR** | | |
| Mild | 5 (2.8) | |
| Moderate | 54 (30.2) | |
| **Seriousness of ADR** | | |
| Serious | 0 (0) | |
| Not Serious | 59 (33) | |
| **Treatment month of onset^†^** | | |
|  | | Mean (10.95) SD (3.56) Median (11) |
| **Treatment month at which adverse event resolved^†^** | | |
|  | Mean (16.18) SD (3.28) Median (16.5) | |
| **Actions taken to manage adverse event potentially caused by cycloserine** | | |
| Counselling | 1 (0.6) | |
| Temporary discontinuation | 2 (1.1) | |
| Ancillary drug added | 54 (30.2) | |
| Not recorded * | 2 (1.1) | |
| **Outcome after action taken for potentially cycloserine induced adverse event** | | |
| Resolved | 56 (31.3) | |
| Not resolved | 2 (1.1) | |
| Not recorded | 1 (0.6) | |

*Data were not available in Electronic Nominal Registration System (ENRS) record of the patient, †Mean, standard deviation and median were taken of both the treatment months at which adverse drug reaction (ADR) occurred and resolved respectively

1. **Nausea and vomiting**

| **Variable** | **n (%)** | |
| --- | --- | --- |
| **Incidence** | 49 (27.4) | |
| **Potential culprit drugs** | | |
| Pyrazinamide | 34 (19) | |
| Levofloxacin | 1 (0.6) | |
| Ethionamide | 4 (2.2) | |
| Para amino salicylic acid | 4 (2.2) | |
| Not recorded * | 6 (3.3) | |
| **Severity of adverse event** | | |
| Mild | 20 (11.2) | |
| Moderate | 28 (15.6) | |
| Not recorded | 1 (0.6) | |
| **Seriousness of adverse event** | | |
| Serious | 1 (0.6) | |
| Not serious | 47 (26.3) | |
| Not recorded | 1 (0.6) | |
| **Treatment month of adverse event onset^†^** | | |
|  | | Mean (7.7) SD (6.3) Median (5.5) |
| **Treatment month at which adverse event resolved^†^** | | |
|  | Mean (8.5) SD (6.5) Median (7) | |
| **Actions taken to manage adverse event potentially caused by pyrazinamide** | | |
| Counselling | 3 (1.7) | |
| Temporary discontinuation | 1 (0.6) | |
| Ancillary drug added | 23 (12.8) | |
| Dose reduction | 6 (3.3) | |
| Not recorded | 1 (0.6) | |
| **Actions taken to manage adverse event potentially caused by levofloxacin** | | |
| Ancillary drug added | 1 (0.6) | |
| **Actions taken to manage adverse event potentially caused by ethionamide** | | |
| Temporary discontinuation | 1 (0.6) | |
| Ancillary drug added | 2 (1.1) | |
| Dose reduction | 1 (0.6) | |
| **Actions taken to manage adverse event potentially caused by para amino salicylic acid** | | |
| Temporary discontinuation | 1 (0.6) | |
| Ancillary drug added | 2 (1.1) | |
| Dose reduction | 1 (0.6) | |
| **Actions taken to manage adverse event potentially caused by non-recorded drug** | | |
| Ancillary drug added | 6 (3.3) | |
| **Outcome after action taken for adverse event potentially induced by pyrazinamide** | | |
| Resolved | 28 (15.6) | |
| Not resolved | 4 (2.2) | |
| Not recorded | 2 (1.1) | |
| **Outcome after action taken for adverse event potentially induced by levofloxacin** | | |
| Resolved | 1 (0.6) | |
| **Outcome after action taken for adverse event potentially induced by ethionamide** | | |
| Resolved | 4 (2.2) | |
| **Outcome after action taken for adverse event potentially induced by para amino salicylic** | | |
| Resolved | 4 (2.2) | |
| **Outcome after action taken for adverse event with no documented culprit drug** | | |
| Resolved | 6 (3.3) | |

*Data were not available in Electronic Nominal Registration System (ENRS) record of the patient, †Mean, standard deviation and median were taken of both the treatment months at which adverse drug reaction (ADR) occurred and resolved respectively

1. **Arthralgia**

| **Variable** | **n (%)** | |
| --- | --- | --- |
| **Incidence** | 49 (27.4) | |
| **Potential culprit drugs** | | |
| Pyrazinamide | 47 (26.3) | |
| Levofloxacin | 1 (0.6) | |
| Not recorded * | 1 (0.6) | |
| **Severity of adverse event** | | |
| Mild | 26 (14.5) | |
| Moderate | 23 (12.8) | |
| **Seriousness of adverse event** | | |
| Serious | 1 (0.6) | |
| Not serious | 48 (26.8) | |
| **Treatment month of adverse event** | | |
|  | | Mean (7) SD (5.8) Median (5) |
| **Treatment month at which adverse event resolved^†^** | | |
|  | Mean (8.5) SD (6.4) Median (7) | |
| **Actions taken to manage adverse event potentially caused by pyrazinamide** | | |
| Temporary discontinuation | 2 (1.1) | |
| Ancillary drug added | 41 (22.9) | |
| Dose reduction | 3 (1.7) | |
| Not recorded | 1 (0.6) | |
| **Actions taken to manage adverse event potentially caused by levofloxacin** | | |
| Ancillary drug added | 1 (0.6) | |
| **Actions taken to manage adverse event potentially caused by non-recorded drug** | | |
| Ancillary drug added | 1 (0.6) | |
| **Outcome after action taken for adverse event potentially induced by pyrazinamide** | | |
| Resolved | 45 (25.1) | |
| Not resolved | 2 (1.1) | |
| **Outcome after action taken for adverse event potentially induced by levofloxacin** | | |
| Not resolved | 1 (0.6) | |
| **Outcome after action taken for adverse event with no documented culprit** | | |
| Resolved | 1 (0.6) | |

*Data were not available in Electronic Nominal Registration System (ENRS) record of the patient, †Mean, standard deviation and median were taken of both the treatment months at which adverse drug reaction (ADR) occurred and resolved respectively

1. **Hearing disturbance**

| **Variable** | **n (%)** | |
| --- | --- | --- |
| **Incidence** | 16 (8.9) | |
| **Potential culprit drug** | | |
| Amikacin | 15 (8.4) | |
| Cycloserine | 1 (0.6) | |
| **Severity of adverse event** | | |
| Mild | 3 (1.7) | |
| Moderate | 13 (7.3) | |
| **Seriousness of adverse event** | | |
| Serious | 8 (4.5) | |
| Not serious | 8 (4.5) | |
| **Treatment month of adverse event onset^†^** | | |
|  | | Mean (6.9) SD (4.1) Median (6.5) |
| **Treatment month at which adverse event resolved^†^** | | |
|  | Mean (7) SD (1.6) Median (8) | |
| **Actions taken to manage adverse event potentially caused by amikacin** | | |
| Temporary discontinuation | 5 (2.8) | |
| Dose reduction | 10 (5.6) | |
| **Actions taken to manage adverse event potentially caused by cycloserine** | | |
| Temporary discontinuation | 1 (0.6) | |
| **Outcome after action taken for adverse event potentially induced by amikacin** | | |
| Resolved | 12 (6.7) | |
| Not resolved | 2 (1.1) | |
| Not recorded ^*^ | 1 (0.6) | |
| **Outcome after action taken for adverse event potentially induced by cycloserine** | | |
| Resolved | 1 (0.6) | |

*Data were not available in Electronic Nominal Registration System (ENRS) record of the patient, †Mean, standard deviation and median were taken of both the treatment months at which adverse drug reaction (ADR) occurred and resolved respectively

1. **Psychosis**

| **Variable** | **n (%)** | |
| --- | --- | --- |
| **Incidence** | 13 (7.3) | |
| **Potential culprit drug** | | |
| Cycloserine | 13 (7.3) | |
| **Severity of adverse event** | | |
| Mild | 2 (1.1) | |
| Moderate | 10 (5.6) | |
| Severe | 1 (0.6) | |
| **Seriousness of adverse event** | | |
| Serious | 4 (2.2) | |
| Not serious | 9 (5) | |
| **Treatment month of adverse event onset^†^** | | |
|  | | Mean (9) SD (4.9) Median (11) |
| **Treatment month at which adverse event resolved^†^** | | |
|  | Mean (11.5) SD (5) Median (13.5) | |
| **Actions taken to manage adverse event potentially caused by cycloserine** | | |
| Temporary discontinuation | 4 (2.2) | |
| Ancillary drug added | 8 (4.5) | |
| Dose reduction | 1 (0.6) | |
| **Outcome after action taken for adverse event potentially induced by cycloserine** | | |
| Resolved | 7 (3.9) | |
| Not resolved | 5 (2.8) | |
| Not recorded | 1 (0.6) | |

*Data were not available in Electronic Nominal Registration System (ENRS) record of the patient, †Mean, standard deviation and median were taken of both the treatment months at which adverse drug reaction (ADR) occurred and resolved respectively

1. **Dyspnea**

| **Variable** | **n (%)** | |
| --- | --- | --- |
| **Incidence** | 12 (6.7) | |
| **Potential culprit drug** | | |
| Pyrazinamide | 2 (1.1) | |
| Para amino salicylic acid | 1 (0.6) | |
| Not recorded * | 9 (5.1) | |
| **Severity of adverse event** | | |
| Mild | 7 (3.9) | |
| Moderate | 3 (1.7) | |
| Not recorded | 2 (1.1) | |
| **Seriousness of adverse event** | | |
| Not serious | 10 (5.6) | |
| Not recorded | 2 (1.1) | |
| **Treatment month of adverse event onset^†^** | | |
|  | | Mean (6.8) SD (5.7) Median (4.5) |
| **Treatment month at which adverse event resolved^†^** | | |
|  | Mean (8.1) SD (5.8) Median (6.5) | |
| **Actions taken to manage adverse event potentially caused by pyrazinamide** | | |
| Ancillary drug added | 2 (1.1) | |
| **Actions taken to manage adverse event potentially caused by para amino salicylic acid** | | |
| Ancillary drug added | 1 (0.6) | |
| **Actions taken to manage adverse event potentially caused by non-recorded drug** | | |
| Ancillary drug added | 6 (3.4) | |
| No action | 1 (0.6) | |
| Not recorded | 2 (1.1) | |
| **Outcome after action taken for adverse event potentially induced by pyrazinamide** | | |
| Resolved | 2 (1.1) | |
| **Outcome after action taken for adverse event potentially induced by para amino salicylic acid** | | |
| Resolved | 1 (0.6) | |
| **Outcome after action taken for adverse event potentially induced by non-recorded drug** | | |
| Resolved | 6 (3.3) | |
| Not recorded | 3 (1.7) | |

*Data were not available in Electronic Nominal Registration System (ENRS) record of the patient, †Mean, standard deviation and median were taken of both the treatment months at which adverse drug reaction (ADR) occurred and resolved respectively

1. **Anorexia**

| **Variable** | **n (%)** | |
| --- | --- | --- |
| **Incidence** | 11 (6.1) | |
| **Potential culprit drugs** | | |
| Pyrazinamide | 2 (1.1) | |
| Levofloxacin | 1 (0.6) | |
| Ethionamide | 1 (0.6) | |
| Para amino salicylic acid | 1 (0.6) | |
| Not recorded * | 6 (3.3) | |
| **Severity of adverse event** | | |
| Mild | 7 (3.9) | |
| Moderate | 3 (1.7) | |
| Not recorded | 1 (0.6) | |
| **Seriousness of adverse event** | | |
| Not serious | 10 (5.6) | |
| Not recorded | 1 (0.6) | |
| **Treatment month of adverse event onset^†^** | | |
|  | | Mean (7.2) SD (5.6) Median (6) |
| **Treatment month at which adverse event resolved^†^** | | |
|  | Mean (24.8) SD (36.6) Median (10.5) | |
| **Actions taken to manage adverse event potentially caused by pyrazinamide** | | |
| Ancillary drug added | 2 (1.1) | |
| **Actions taken to manage adverse event potentially caused by levofloxacin** | | |
| Ancillary drug added | 1 (0.6) | |
| **Actions taken to manage adverse event potentially caused by ethionamide** | | |
| Dose reduction | 1 (0.6) | |
| **Actions taken to manage adverse event potentially caused by para amino salicylic acid** | | |
| Ancillary drug added | 1 (0.6) | |
| **Actions taken to manage adverse event potentially caused by non-recorded drug** | | |
| Counselling | 3 (1.7) | |
| No action | 1 (0.6) | |
| Not recorded | 2 (1.1) | |
| **Outcome after action taken for adverse event potentially induced by pyrazinamide** | | |
| Resolved | 2 (1.1) | |
| **Outcome after action taken for adverse event potentially induced by levofloxacin** | | |
| Not resolved | 1 (0.6) | |
| **Outcome after action taken for adverse event potentially induced by ethionamide** | | |
| Resolved | 1 (0.6) | |
| **Outcome after action taken for adverse event potentially induced by para amino salicylic acid** | | |
| Resolved | 1 (0.6) | |
| **Outcome after action taken for adverse event potentially induced by non-recorded drug** | | |
| Resolved | 4 (2.2) | |
| Not resolved | 1 (0.6) | |
| Not recorded | 1 (0.6) | |

*Data were not available in Electronic Nominal Registration System (ENRS) record of the patient, †Mean, standard deviation and median were taken of both the treatment months at which adverse drug reaction (ADR) occurred and resolved respectively

1. **Peripheral neuropathy**

| **Variable** | **n (%)** | |
| --- | --- | --- |
| **Incidence** | 9 (5) | |
| **Potential culprit drugs** | | |
| Pyrazinamide | 2 (1.1) | |
| Levofloxacin | 1 (0.6) | |
| Cycloserine | 5 (2.8) | |
| Para amino salicylic acid | 1 (0.6) | |
| **Severity of adverse event** | | |
| Mild | 2 (1.1) | |
| Moderate | 7 (3.9) | |
| **Seriousness of adverse event** | | |
| Serious | 1 (0.6) | |
| Not serious | 8 (4.5) | |
| **Treatment month of adverse event onset^†^** | | |
|  | | Mean (10.2) SD (6.5) Median (8) |
| **Treatment month at which adverse event resolved^†^** | | |
|  | Mean (9.6) SD (5.7) Median (9) | |
| **Actions taken to manage adverse event potentially caused by pyrazinamide** | | |
| Temporary discontinuation | 1 (0.6) | |
| Ancillary drug added | 1 (0.6) | |
| **Actions taken to manage adverse event potentially caused by levofloxacin** | | |
| Ancillary drug added | 1 (0.6) | |
| **Actions taken to manage adverse event potentially caused by cycloserine** | | |
| Temporary discontinuation | 1 (0.6) | |
| Ancillary drug added | 3 (1.7) | |
| Dose reduction | 1 (0.6) | |
| **Actions taken to manage adverse event potentially caused by para amino salicylic acid** | | |
| Temporary discontinuation | 1 (0.6) | |
| **Outcome after action taken for adverse event potentially induced by pyrazinamide** | | |
| Resolved | 2 (1.1) | |
| **Outcome after action taken for adverse event potentially induced by levofloxacin** | | |
| Resolved | 1 (0.6) | |
| **Outcome after action taken for adverse event potentially induced by cycloserine** | | |
| Resolved | 5 (2.8) | |
| **Outcome after action taken for adverse event potentially induced by para amino salicylic** | | |
| Not resolved | 1 (0.6) | |

*Data were not available in Electronic Nominal Registration System (ENRS) record of the patient, †Mean, standard deviation and median were taken of both the treatment months at which adverse drug reaction (ADR) occurred and resolved respectively

1. **Headache**

| **Variable** | **n (%)** | |
| --- | --- | --- |
| **Incidence** | 7 (3.9) | |
| **Potential culprit drugs** | | |
| Pyrazinamide | 5 (2.8) | |
| Para amino salicylic acid | 1 (0.6) | |
| Not recorded * | 1 (0.6) | |
| **Severity of adverse event** | | |
| Mild | 4 (2.2) | |
| Moderate | 3 (1.7) | |
| **Seriousness of adverse event** | | |
| Not serious | 7 (3.9) | |
| **Treatment month of adverse event onset^†^** | | |
|  | | Mean (5.1) SD (2.9) Median (4) |
| **Treatment month at which adverse event resolved^†^** | | |
|  | Mean (4.7) SD (1.5) Median (4) | |
| **Actions taken to manage adverse event potentially caused by pyrazinamide** | | |
| Ancillary drug added | 5 (2.8) | |
| **Actions taken to manage adverse event potentially caused by para amino salicylic acid** | | |
| Ancillary drug added | 1 (0.6) | |
| **Actions taken to manage adverse event potentially caused by non-recorded drug** | | |
| No action | 1 (0.6) | |
| **Outcome after action taken for adverse event potentially induced by pyrazinamide** | | |
| Resolved | 5 (2.8) | |
| **Outcome after action taken for adverse event potentially induced by para amino salicylic acid** | | |
| Resolved | 1 (0.6) | |
| **Outcome after action taken for adverse event potentially induced by non-recorded drug** | | |
| Resolved | 1 (0.6) | |

*Data were not available in Electronic Nominal Registration System (ENRS) record of the patient, †Mean, standard deviation and median were taken of both the treatment months at which adverse drug reaction (ADR) occurred and resolved respectively

1. **Dizziness and vertigo**

| **Variable** | **n (%)** | |
| --- | --- | --- |
| **Incidence** | 7 (3.9) | |
| **Potential culprit drugs** | | |
| Pyrazinamide | 2 (1.1) | |
| Levofloxacin | 1 (0.6) | |
| Cycloserine | 2 (1.1) | |
| Not recorded * | 2 (1.1) | |
| **Severity of adverse event** | | |
| Mild | 4 (2.2) | |
| Moderate | 3 (1.7) | |
| **Seriousness of adverse event** | | |
| Not serious | 7 (3.9) | |
| **Treatment month of adverse event onset^†^** | | |
|  | | Mean (12.7) SD (7) Median (13) |
| **Treatment month at which adverse event resolved^†^** | | |
|  | Mean (12) SD (7.8) Median (7) | |
| **Actions taken to manage adverse event potentially caused by pyrazinamide** | | |
| Counselling | 1 (0.6) | |
| Ancillary drug added | 1 (0.6) | |
| **Actions taken to manage adverse event potentially caused by levofloxacin** | | |
| Ancillary drug added | 1 (0.6) | |
| **Actions taken to manage adverse event potentially caused by cycloserine** | | |
| Ancillary drug added | 2 (1.1) | |
| **Actions taken to manage adverse event potentially caused by non-recorded drug** | | |
| Ancillary drug added | 2 (1.1) | |
| **Outcome after action taken for adverse event potentially induced by pyrazinamide** | | |
| Resolved | 2 (1.1) | |
| **Outcome after action taken for adverse event potentially induced by levofloxacin** | | |
| Resolved | 1 (0.6) | |
| **Outcome after action taken for adverse event potentially induced by cycloserine** | | |
| Resolved | 1 (0.6) | |
| Not resolved | 1 (0.6) | |
| **Outcome after action taken for adverse event potentially induced by non-recorded drug** | | |
| Resolved | 2 (1.1) | |

*Data were not available in Electronic Nominal Registration System (ENRS) record of the patient, †Mean, standard deviation and median were taken of both the treatment months at which adverse drug reaction (ADR) occurred and resolved respectively

1. **Gastritis**

| **Variable** | **n (%)** | |
| --- | --- | --- |
| **Incidence** | 6 (3.4) | |
| **Potentially culprit drug** | | |
| Pyrazinamide | 6 (3.4) | |
| **Severity of adverse event** | | |
| Mild | 4 (2.2) | |
| Moderate | 2 (1.1) | |
| **Seriousness of adverse event** | | |
| Not Serious | 6 (3.4) | |
| **Treatment month of adverse event onset^†^** | | |
|  | | Mean (16.7) SD (2.1) Median (17) |
| **Treatment month at which adverse event resolved^†^** | | |
|  | Mean (17) SD (2.2) Median (18) | |
| **Actions taken to manage adverse event potentially caused by pyrazinamide** | | |
| Temporary discontinuation | 2 (1.1) | |
| Ancillary drug added | 4 (2.2) | |
| **Outcome after action taken for adverse event potentially induced by pyrazinamide** | | |
| Resolved | 6 (3.4) | |

*Data were not available in Electronic Nominal Registration System (ENRS) record of the patient, †Mean, standard deviation and median were taken of both the treatment months at which adverse drug reaction (ADR) occurred and resolved respectively

1. **Rash and pruritus**

| **Variable** | **n (%)** | |
| --- | --- | --- |
| **Incidence** | 6 (3.4) | |
| **Potential culprit drug** | | |
| Pyrazinamide | 2 (1.1) | |
| Amikacin | 1 (0.6) | |
| Not recorded * | 3 (1.7) | |
| **Severity of adverse event** | | |
| Mild | 3 (1.7) | |
| Moderate | 3 (1.7) | |
| **Seriousness of adverse event** | | |
| Not serious | 6 (3.4) | |
| **Treatment month of adverse event onset^†^** | | |
|  | | Mean (7) SD (5.6) Median (5.5) |
| **Treatment month at which adverse event resolved^†^** | | |
|  | Mean (8.3) SD (6.7) Median (6) | |
| **Actions taken to manage adverse event potentially caused by pyrazinamide** | | |
| Temporary discontinuation | 1 (0.6) | |
| Ancillary drug added | 1 (0.6) | |
| **Actions taken to manage adverse event potentially caused by amikacin** | | |
| Dose reduction | 1 (0.6) | |
| **Actions taken to manage adverse event potentially caused by non-recorded drug** | | |
| Ancillary drug added | 2 (1.1) | |
| Not recorded | 1 (0.6) | |
| **Outcome after action taken for adverse event potentially induced by pyrazinamide** | | |
| Resolved | 2 (1.1) | |
| **Outcome after action taken for adverse event potentially induced by amikacin** | | |
| Resolved | 1 (0.6) | |
| **Outcome after action taken for adverse event potentially induced by non-recorded drug** | | |
| Resolved | 2 (1.1) | |
| Not recorded | 1 (0.6) | |

*Data were not available in Electronic Nominal Registration System (ENRS) record of the patient, †Mean, standard deviation and median were taken of both the treatment months at which adverse drug reaction (ADR) occurred and resolved respectively
